# First participant diagnosed with Creutzfeldt-Jakob disease in the population-based Rotterdam Study was classified with mild cognitive impairment

**DOI:** 10.1136/bcr-2020-235509

**Published:** 2021-03-29

**Authors:** Hata Karamujić-Čomić, Annemieke J M Rozemuller, M Arfan Ikram, Cornelia M van Duijn

**Affiliations:** 1 Department of Epidemiology, Erasmus MC, University Medical Center, Rotterdam, The Netherlands; 2 National Prion Disease Registry, Department of Epidemiology, Erasmus MC, University Medical Center, Rotterdam, The Netherlands; 3 Department of Pathology, Amsterdam Neuroscience, Amsterdam UMC, VU University Medical Center, Amsterdam, The Netherlands; 4 Department of Pathology, UMC Utrecht, Utrecht, The Netherlands; 5 Clinical Trial Service Unit and Epidemiological Studies Unit, Nuffield Department of Population Health, University of Oxford, Oxford, UK

**Keywords:** variant creutzfeld-jakob disease, dementia, alzheimer's type, memory disorders

## Abstract

Sporadic Creutzfeldt-Jakob disease (sCJD) is a rare, fatal, neurodegenerative disease caused by accumulation of abnormally folded prion protein. sCJD can have a long asymptomatic incubation period, with little known about this period. We describe the first-ever participant within the population-based Rotterdam Study diagnosed with sCJD. We retrieved clinical data from both the population-based Rotterdam Study and the National Prion Disease Registry. In 2011, a female participant of the Rotterdam Study was diagnosed with probable sCJD and registered into the Registry. Four months earlier, she was classified as having mild cognitive impairment based on assessment in the Rotterdam Study. Clinical deterioration was rapid, with the patient dying 7 months after the research centre visit. Postmortem brain autopsy confirmed the diagnosis of sCJD. In conclusion, we describe the first case diagnosed with sCJD who during diagnostic workup for sCJD was classified as having mild cognitive impairment in a population-based cohort study.

## Background

Creutzfeldt-Jakob disease (CJD) is a rare, fatal, neurodegenerative disease caused by accumulation of abnormally folded prion protein. It is the most common prion disease and accounts for approximately 90% of all prion disease cases.^1^ It mostly presents with rapidly progressive dementia, ataxia and myoclonus. The clinical course differs according to subtype of CJD, that is, sporadic, iatrogenic, familial and variant.^2 3^ The sporadic form of CJD (sCJD) is the most common form of CJD and in most cases patients die within months.^4^ Currently, routine diagnostic investigations include brain MRI, electroencephalography (EEG) and cerebrospinal fluid (CSF) analyses.^5 6^ Typical brain MRI findings for sCJD are high signal in specific regions: putamen or caudate, or high signal in at least two cortical regions (temporal, parietal, occipital). Characteristic EEG findings for sCJD are periodic biphasic and triphasic sharp wave complexes. In CSF analyses, typical abnormalities include positive 14-3-3 protein, high total tau protein (t-tau), high ratio between phosphorylated tau (p-tau) and t-tau, high S100 protein and high neuron-specific enolase (NSE). The real-time quaking-induced conversion test in CSF is a relatively new diagnostic test which is highly sensitive and specific for sCJD.^7^ Yet, postmortem brain autopsy remains the standard for definite confirmation of CJD. Postmortem brain autopsy can provide further classification of the molecular subtype of sCJD.^8 9^ sCJD can have an asymptomatic incubation period lasting for decades,^3^ but little is known about this long disease-free period before symptom onset.

Here, we describe a participant from the population-based Rotterdam Study who developed sCJD. The study has a longitudinal design including multiple visits at the research centre with extensive investigations, which provides us a unique opportunity to detail the preclinical phase of sCJD. In this article, we describe the clinical history of this patient, retrieved from both the Rotterdam Study and the National Prion Disease Registry.

## Case presentation

Our case was a 76-year-old woman with a medical history of hyperthyroidism, varices, recurrent urinary tract infections, paroxysmal atrial fibrillation, cholecystectomy and uterus extirpation. She was married and had worked as nurse. With regard to the patient’s participation in the population-based follow-up study, the Rotterdam Study, our case entered this study in 1991, and participated up to the fifth examination in November 2010.

From May 2010, she started to develop symptoms of forgetfulness, dizziness, steerlessness in the right leg, and a heavy feeling in the head and eyes. Over a period of a few months, there was fast progression and she had difficulties with her balance and walked unstable. She also had an increased fall tendency. Besides, she had a feeling of pressure in the left ear, visual problems and sometimes diplopia. The patient was seen by an ophthalmologist and an otorhinolaryngologist, both specialists found no abnormalities. Regarding the typical symptoms for sCJD, over a period of months, she had progressive cognitive decline, cerebellar signs, pyramidal signs and extrapyramidal signs. The patient was registered at the Dutch National Prion Disease Registry in March 2011, where according to protocol an in-depth study was conducted on the medical history. There was no iatrogenic history explaining the CJD. During the extensive interview of the family, no evidence for a positive family history emerged.

## Investigations

### Research visit examinations

During the fourth examination of Rotterdam Study in May 2004, at age 70 years, cognitive tests were performed and showed no abnormalities, with scores for the Mini-Mental State Examination (MMSE) 30 out of 30 and good scores for the other cognitive tests, as described in table 1. Furthermore, routine locomotor symptom screening showed no abnormalities. During the fifth examination round in November 2010, at age 77 years, the participant reported that she suffered from cognitive complaints and cognitive testing showed abnormalities. The MMSE score was 22 out of 30 and compared with the previous examinations she had lower scores on the Word fluency task, Purdue Pegboard Test, words learning test and Stroop test. Detailed information on the results of the cognitive tests is described in table 1. Based on the presence of subjective cognitive complaints, the presence of objective cognitive impairment, and the absence of dementia, she was classified in the Rotterdam Study as having mild cognitive impairment.^10^ During locomotor symptom screening we found no dysarthria, no tremor, no abnormalities during finger tapping, normotonic arms and legs, and no dysmetria. At the standing and gait examinations, she was able to stand stable on two legs, but not in tandem position. She was able to walk independently, though with a slight unstable gait pattern. A non-contrast brain MRI was performed (in November 2010) as part of the research protocol and was compared with the brain MRI from other Rotterdam Study participants aged 70–96 years (median age of 77 years). This revealed that she had a smaller volume of the right and left hippocampus, right thalamus, left amygdala and brainstem. She had a larger volume of CSF, right and left nucleus accumbens, and right putamen than expected for her age. There were no changes on the brain MRI at the basal ganglia, as verified a posteriori by the CJD expert centre at the University of Edinburgh.

**Table 1:** Results of the cognitive tests before and at diagnosis of the Creutzfeldt-Jakob disease case

Cognitive test (unit)	Follow-up in 2004	Follow-up in 2010
Mini-mental state examination (score/total)	30/30	22/30
Letter digit test (score/total)	25/25	20/20
Fluency task (score/total)	25/25	19/21
Purdue Pegboard test right hand	14	10
Purdue Pegboard test left hand	13	11
Purdue Pegboard test both hand pairs	11	9
15 words learning test trial 1 (no. correct)	6	2
15 words learning test trial 2 (no. correct)	6	4
15 words learning test trial 3 (no. correct)	10	6
15 words learning test trial delayed recall (no. correct)	7	1
15 words learning test recognition task (no. positive correct)	15	15
Stroop test 1 (time (seconds), failures)	14.6, 0	15.5, 0
Stroop test 2 (time (seconds), failures)	18.6, 0	21.2, 0
Stroop test 3 (time (seconds), failures)	48.7, 1	54.6, 14

### Clinical investigations

**Figure 1:**
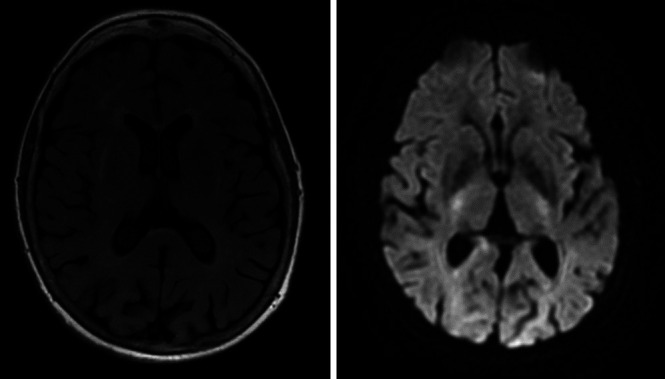
Axial brain MRI images showing multiple white matter hyperintensities; left: fluid-attenuated inversion recovery brain MRI sequence; right: diffusion-weighted imaging brain MRI sequence.

Brain MRI which was performed in October 2010—around 4 weeks before the brain MRI in the Rotterdam Study research centre—showed intracerebral multiple white matter hyperintensities, which were confluent periventricular. These white matter hyperintensities were most likely of vascular origin. Also, slightly wider peripheral and central CSF spaces were seen on brain MRI. The MRI did not show high signal in putamen or caudate. MRI images are shown in figure 1. Furthermore, an EEG, which was performed in March 2011, showed sufficient fast activity and showed occipital rhythm and diffuse rhythm. Diffuse slow activity was frequently seen as well. The EEG did not show sharp wave complexes. Analysis of the CSF, performed in March 2011, showed a weakly positive 14-3-3 protein, elevated t-tau (4800 ng/L, normal <350), with normal p-tau (72 ng/L, normal <85), elevated S100 protein (8.2 µg/L, normal <3.9) and elevated NSE (20.8 µg/L, normal <17.5). The MMSE score at that time was still 25 out of 30. The patient was heterozygous for codon 129 from the prion protein gene (PRNP; genotype MV) and for the apolipoprotein E gene (genotype epsilon 2/epsilon 3).

## Differential diagnosis

Several differential diagnoses were considered and evaluated. Table 2 gives an overview of the differential diagnoses and on how they were ruled out. Based on the diagnostic criteria for sCJD,^11^ the patient was diagnosed with probable sCJD. The criteria which were met were the presence of progressive cognitive impairment, visual problems, cerebellar problems, pyramidal signs and extrapyramidal signs and positive 14-3-3 protein in CSF.

**Table 2:** Overview of the differential diagnoses and performed investigations to rule them out

Differential diagnosis ruled out	Investigations
Progressive supranuclear palsy	Clinical features, brain MRI
Vestibular schwannoom	Brain MRI
Brain metastases	Brain MRI
Alzheimer’s disease	Clinical features, brain MRI
Stroke	Brain MRI
Cerebral amyloid angiopathy	Brain MRI
Electrolyte abnormalities	Elektrolytes panel

## Outcome and follow-up

**Figure 2:**
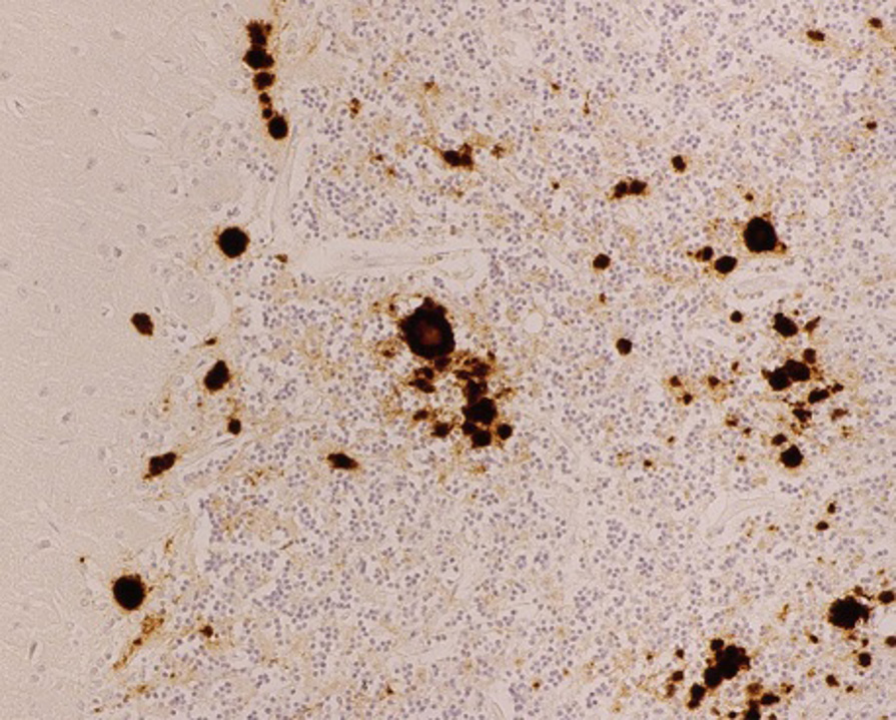
Microscopic pathology demonstrating kuru-type plaques in the cerebellum.

Our case was diagnosed as a probable sCJD case at the age of 77 years. The patient died in June 2011, 3 months after the diagnosis sCJD was clinically suspected. Postmortem brain autopsy confirmed the diagnosis. Pathological investigation showed widespread spongiosis and gliosis. Prion protein immunohistochemistry showed synaptic staining, mainly in the deeper layers with most spongiosis, plaque-like deposits and compact deposits. In the cerebellar cortex spongiosis and frequent periodic acid–Schiff (PAS)-positive plaques (kuru-type plaques) and deposits were seen, as shown in figure 2. The cerebellar grey matter also showed some PAS-positive plaques. Other parts of the brain, including the cerebral cortex, basal nuclei, hippocampus, amygdala, thalamus and brain stem, showed spongiosis and some PAS-positivity. PRNP gene analysis revealed no mutations. Based on the pathological alterations, the final histopathological diagnosis was sCJD type MV2K.^8 9 12^

## Discussion

sCJD is the most common form of CJD.^4^ We report the first case diagnosed with sCJD who was enrolled in a population-based cohort study and was classified as having mild cognitive impairment during the diagnostic workup for sCJD.

sCJD is a fatal neurological disease with a large variety in symptom onset and progression. Rapid progressive cognitive decline leading to dementia is often the first clinical manifestation of sCJD. We describe the first case diagnosed with sCJD in the population-based Rotterdam Study and for whom prospective epidemiological data is available. During the patient’s last visit at our research centre, she had subjective cognitive complaints and, based on cognitive examinations, she also had objective cognitive impairment. Therefore, she was classified within the study as having mild cognitive impairment. At the same time, the patient was suffering from several other neurological symptoms and was referred to the neurology clinic for diagnostic workup. Given that sCJD is a progressive disease in which symptoms are known to progress rapidly, the cognitive decline observed during her final visit to our research centre was not unexpected. However, since she did not yet fulfil the criteria for dementia, she was classified as having mild cognitive impairment. This was beyond our expectations, as the visit at our research centre was during the early clinical stage of sCJD, that is, 4 months before diagnosis of sCJD. This shows that the symptom onset of cognitive impairment in our sCJD case was subtle.^13^ Moreover, the MMSE score a few months before death showed subtle cognitive impairment. The recognition of sCJD is often a diagnostic challenge.

sCJD cases with the molecular subtype MV2K have a long clinical course and usually present with early-onset ataxia. This was seen in our case as well, as dominant symptoms were the difficulties with balance and unstable walking. Further, in cases with this molecular subtype, sharp wave complexes on EEG are infrequently seen, as was the case in our patient. It is of interest that the patient had a late symptom onset. The age of onset of the cognitive problems in our case was 76 years, which is higher than the mean age of onset for sCJD.^9 14^ The mean age of onset in the Netherlands is 66 years.^15^ In sCJD cases with the molecular subtype MV2K, the median age of onset is known to be 65 years (range 36–83 years).^9 16^ Our case can be considered as a relatively late-onset case of sCJD. A possible explanation for the late onset of mild cognitive impairment symptoms is that our case was high-functioning before symptom onset, which could have provided cognitive protection.

In conclusion, we describe the first case diagnosed with sCJD who during diagnostic workup for sCJD was classified as having mild cognitive impairment in a population-based cohort study.

**Learning points**

- The sporadic form of Creutzfeldt-Jakob disease (sCJD) is the most common prion disease, which is a fatal, neurodegenerative disease caused by accumulation of abnormally folded prion protein.
- As the symptom onset of sCJD can be subtle, the diagnosis of sCJD can be a diagnostic challenge, especially in the early clinical stage of the disease.
- In sCJD cases with the molecular subtype MV2K, the median age of onset is 65 years with a large range of age of onset. Furthermore, this subtype of sCJD has a long clinical course.
- We describe the first-ever participant who was enrolled in the population-based Rotterdam Study and who was diagnosed with sCJD.

## References

[1] Creutzfeldt-Jakob Disease International Surveillance Network. Cjd surveillance data 1993-2018. Available: http://www.eurocjd.ed.ac.uk/surveillance%20data%201.html

[2] Jansen C, Parchi P, Capellari S, et al. Human prion diseases in the Netherlands (1998-2009): clinical, genetic and molecular aspects. PLoS One 2012;7:e36333. 10.1371/journal.pone.0036333 22558438 PMC3340342

[3] Uttley L, Carroll C, Wong R, et al. Creutzfeldt-Jakob disease: a systematic review of global incidence, prevalence, infectivity, and incubation. Lancet Infect Dis 2020;20:e2–10. 10.1016/S1473-3099(19)30615-2 31876504

[4] Zerr I, Parchi P. Sporadic Creutzfeldt-Jakob disease. Handb Clin Neurol 2018;153:155–74. 10.1016/B978-0-444-63945-5.00009-X 29887134

[5] Collins S, Boyd A, Fletcher A, et al. Recent advances in the pre-mortem diagnosis of Creutzfeldt-Jakob disease. J Clin Neurosci 2000;7:195–202. 10.1054/jocn.1999.0191 10833615

[6] Zanusso G, Monaco S, Pocchiari M, et al. Advanced tests for early and accurate diagnosis of Creutzfeldt-Jakob disease. Nat Rev Neurol 2016;12:325–33. 10.1038/nrneurol.2016.65 27174240

[7] Green AJE. Rt-Quic: a new test for sporadic CJD. Pract Neurol 2019;19:49–55. 10.1136/practneurol-2018-001935 30282760 PMC6580883

[8] Parchi P, Giese A, Capellari S, et al. Classification of sporadic Creutzfeldt-Jakob disease based on molecular and phenotypic analysis of 300 subjects. Ann Neurol 1999;46:224–33. 10.1002/1531-8249(199908)46:2&lt;224::AID-ANA12&gt;3.0.CO;2-W 10443888

[9] Parchi P, Strammiello R, Notari S, et al. Incidence and spectrum of sporadic Creutzfeldt-Jakob disease variants with mixed phenotype and co-occurrence of PrPSc types: an updated classification. Acta Neuropathol 2009;118:659–71. 10.1007/s00401-009-0585-1 19718500 PMC2773124

[10] de Bruijn RFAG, Akoudad S, Cremers LGM, et al. Determinants, MRI correlates, and prognosis of mild cognitive impairment: the Rotterdam study. J Alzheimers Dis 2014;42 Suppl 3:S239–49. 10.3233/JAD-132558 24825566

[11] Zerr I, Kallenberg K, Summers DM, et al. Updated clinical diagnostic criteria for sporadic Creutzfeldt-Jakob disease. Brain 2009;132:2659–68. 10.1093/brain/awp191 19773352 PMC2759336

[12] Parchi P, de Boni L, Saverioni D, et al. Consensus classification of human prion disease histotypes allows reliable identification of molecular subtypes: an inter-rater study among surveillance centres in Europe and USA. Acta Neuropathol 2012;124:517–29. 10.1007/s00401-012-1002-8 22744790 PMC3725314

[13] Geschwind MD, Murray K. Differential diagnosis with other rapid progressive dementias in human prion diseases. Handb Clin Neurol 2018;153:371–97. 10.1016/B978-0-444-63945-5.00020-9 29887146

[14] de Silva R, Findlay C, Awad I, et al. Creutzfeldt-Jakob disease in the elderly. Postgrad Med J 1997;73:557–9. 10.1136/pgmj.73.863.557 9373595 PMC2431436

[15] Jansen C, Schuur M, Spliet WGM, et al. Eleven years of autopsy on account of Creutzfeldt-Jakob disease in the Netherlands. Ned Tijdschr Geneeskd 2009;153:A172. 19785859

[16] Krasnianski A, Schulz-Schaeffer WJ, Kallenberg K, et al. Clinical findings and diagnostic tests in the MV2 subtype of sporadic CJD. Brain 2006;129:2288–96. 10.1093/brain/awl123 16720682

